# Real-time intermembrane force measurements and imaging of lipid domain morphology during hemifusion

**DOI:** 10.1038/ncomms8238

**Published:** 2015-05-26

**Authors:** Dong Woog Lee, Kai Kristiansen, Stephen H. Donaldson, Jr., Nicholas Cadirov, Xavier Banquy, Jacob N. Israelachvili

**Affiliations:** 1 Department of Chemical Engineering, University of California, Santa Barbara, California 93106, USA; 2 Canada Research Chair in Bio-inspired Materials and Interfaces, Faculty of Pharmacy, Université de Montréal, C.P. 6128, Succursale Centre Ville, Montréal, Quebec H3C 3J7, Canada; 3 Department of Materials, University of California, Santa Barbara, California 93106, USA

## Abstract

Membrane fusion is the core process in membrane trafficking and is essential for cellular transport of proteins and other biomacromolecules. During protein-mediated membrane fusion, membrane proteins are often excluded from the membrane–membrane contact, indicating that local structural transformations in lipid domains play a major role. However, the rearrangements of lipid domains during fusion have not been thoroughly examined. Here using a newly developed Fluorescence Surface Forces Apparatus (FL-SFA), migration of liquid-disordered clusters and depletion of liquid-ordered domains at the membrane–membrane contact are imaged in real time during hemifusion of model lipid membranes, together with simultaneous force–distance and lipid membrane thickness measurements. The load and contact time-dependent hemifusion results show that the domain rearrangements decrease the energy barrier to fusion, illustrating the significance of dynamic domain transformations in membrane fusion processes. Importantly, the FL-SFA can unambiguously correlate interaction forces and *in situ* imaging in many dynamic interfacial systems.

## Abstract (web summary)

During membrane fusion, lipid bilayers come into direct contact but rearrangements of lipid domains during fusion have not been thoroughly examined. Here the authors observe and correlate membrane morphology, interaction forces and domain rearrangements during hemifusion of two model membranes.

## Graphical abstract

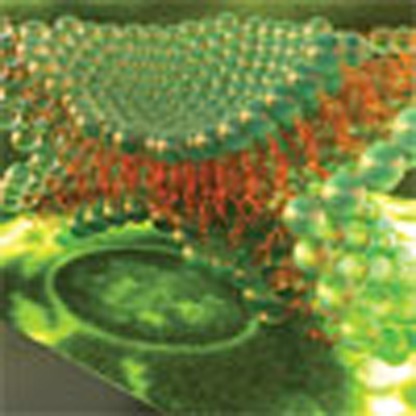

Lipid domains are clusters or two-dimensional aggregates of lipids whose molecular composition differs from the surrounding membrane^1^. One commonly observed lipid domain, the sphingolipid and cholesterol (CHOL)-enriched domain, plays important roles in many biological membrane fusion processes. Lipid domains are associated with protein-binding sites during exo- and endocytosis^2^,^3^, which are essential for transport of protein and vesicle cargo^4^,^5^. In addition, ion channels for electrical signal transduction are localized in lipid domains^6^,^7^. In extracellular processes, lipid domains are known to act as viral gateways or pathogen-binding sites in diseases such as Alzheimer's, bovine spongiform encephalopathy (also known as ‘mad cow disease') and HIV-1 (ref. 8).

Previous studies on combined lipid and protein systems show that lipid domains localize SNARE proteins^2^,^3^,^9^, and the formation of lipid domain/SNARE complexes is essential for lowering the energy barrier to fusion^10^. Other studies on myogenic cells show that lipid domains dynamically cluster and disperse during different stages of fusion, contributing to cell adhesion and plasma membrane union^11^. Furthermore, during fusion, the membrane proteins are eventually excluded from the membrane–membrane contact zone^12^, at which point protein-free intermembrane interactions become significant as also in nonbiological, surfactant membrane fusion processes^13^. Such interactions include van der Waals, steric hydration, electrostatic and hydrophobic interactions that eventually drive the membranes to fuse^13^,^14^. The structural intermediates formed during the fusion process, known as fusion ‘stalks,' have been reported to depend delicately on lipid membrane composition^15^. Although the involvement of lipid domains during biological fusion processes is now well established, their dynamic rearrangements during fusion are yet to be elucidated.

Such domains are seen in reconstituted myelin lipid bilayers extracted from the brain, for example, where the domains are observed to be different in healthy versus pathological (for example, multiple sclerosis) membranes^16^. In this case, as in many other cellular structures, the membranes are planar and closely stacked *in vivo* and therefore strongly interacting with each other across the water spaces. While these domains have not been observed *in vivo*, many experiments have shown correlations between the *in vitro* structures observed in healthy and diseased membranes^16^,^17^. Indeed, many studies of domains focus on model or supported membrane systems to draw (perhaps indirect) correlations to the *in vivo* systems. Experiments on model systems, while inherently nonbiological, can be used to find correlations and insights of fundamental importance.

Therefore, in this work we aim to determine and correlate the membrane morphology, interactions, domain structures and the time dependence of rearrangements within the domains during contact, compression, adhesion and fusion of two model membranes. Using a custom-built Fluorescence Surface Forces Apparatus (FL-SFA, see Methods section and Fig. 1 for a detailed description of the set-up), we measured the interaction forces between supported lipid membranes and simultaneously imaged lipid domains during pressure-induced and protein-free hemifusion, allowing for real-time correlations to be made between the interaction forces, membrane thickness and spatial and temporal domain rearrangements. The results on the model membranes provide mechanistic details of domain rearrangements during membrane fusion, demonstrating that the FL-SFA should find wide utility in correlating fluorescent images with interaction forces during compression and separation of a broad range of materials between confined surfaces.

## Results

### Lipid domain visualization in the FL-SFA

Figure 2a shows the schematic of the bilayer substrates used for the experiments. Briefly, asymmetric lipid bilayers were deposited on freshly cleaved mica surfaces using Langmuir–Blodgett (LB) deposition (see Methods). 1,2-dipalmitoyl-*sn*-glycero-3-phosphoethanolamine (DPPE) was deposited on mica as a supporting first monolayer (Supplementary Fig. 1a). As a second layer, a 1:1:1 mixture of 1,2-dioleoyl-*sn*-glycero-3-phosphocholine (DOPC), Brain sphingomyelin (BSM) and CHOL, with a trace amount (1 wt%) of Texas Red 1,2-Dihexadecanoyl-sn-Glycero-3-Phosphoethanolamine, Triethylammonium Salt (TR-DHPE), was deposited on the DPPE monolayer (Supplementary Fig. 1b), and readily forms lipid domain structures (Supplementary Fig. 1c).

Using the FL-SFA, bilayers containing lipid domains were imaged inside the SFA (Fig. 2 and Supplementary Fig. 2). When the bilayers are positioned far apart (mica–mica separation distance *D*>500 μm), only the upper bilayer is visible (Fig. 2b) because the lower bilayer is out of focus. The dark regions indicate the liquid-ordered phase (L_o_) of lipid bilayers, conventionally referred to as lipid domains, which are rich in BSM and CHOL^18^,^19^. The bright regions, where TR-DHPE is selectively localized, are in the liquid-disordered phase (L_d_) of lipid bilayers and rich in DOPC^18^,^19^. Noncircular and large domains are observed, while others observed circular and smaller domains in similar systems^20^,^21^. The irregular domain shapes observed here are primarily because of the presence of calcium ions in the subphase during the LB deposition, which are known to bind strongly to the bilayer, induce phase separation, presumably make larger and irregularly shaped domains, and also lower the energy barrier to membrane fusion^22^,^23^. The domain size and shape at different lateral pressures (*Π*=5, 15 and 30 mN m^−1^) are shown in Supplementary Fig. 1. When the bilayers are positioned closer to each other (*D*<5 μm), lipid domains (L_o_) in both bilayers are observed (Fig. 2c) along with Newton's interference rings. Here dark, grey and white regions indicate domain–domain overlap between upper and lower bilayers (L_o_–L_o_), isolated domains only in one bilayer (L_o_–L_d_) and no domains in both bilayers (L_d_–L_d_), respectively. By comparing the images of the upper bilayer (Fig. 2b) and both bilayers (Fig. 2c), domains (L_o_) in the lower bilayer can be identified as well (Fig. 2d).

### Force and thickness measurements between hemifusing bilayers

Interaction forces (*F*/*R*) between the bilayers were measured as a function of separation distance (*D*) with simultaneous fluorescence imaging (Fig. 3). Three distinct force runs (FRs) were performed where the bilayers were brought into contact under low compression (*F*/*R*=8 mN m^−1^) and then separated after a contact time (*t*_c_): (i) FR1: *t*_c_=0 min, (ii) FR2: *t*_c_=19 h and (iii) FR3: *t*_c_=0 min, but at a previously hemifused contact region.

The force curve (FR1) shows no hysteresis between approach and separation, and a steric (hard) wall thickness (*D* at *F*/*R*=8 mN m^−1^) similar to the thickness, *T*, of two bilayers (*T*=2*D*_B_). The approach run of FR2 is similar to FR1 with the same steric hard wall thickness; however, during 19 h of contact, *slow* hemifusion of the bilayers is observed. The thickness of two bilayers (*T*=2*D*_B_=8.7 nm) decreases down to one bilayer thickness (*T*=*D*_B_=4.4 nm) over time (Fig. 3b). The thickness decrease was fitted with an exponential decay equation: *T*=C_0_+C_1_·exp(−*t*_c_/*τ*), where C_0_ and C_1_ are constants and exhibit two different regimes (Fig. 3b). In the first regime (*t*_c_<200 min), the thickness decreases with a characteristic time, *τ*, of 56±12 min (±values are the s.d. of at least three different replicates), while the second regime (*t*_c_>200 min) has *τ*=510±100 min. The first regime is governed by the compression and thinning of the outer monolayer, while the second regime is likely related to the hydrophobic interaction and hemifusion of the lipid bilayers.

The approach curve (FR2) was fitted to a previously developed interaction potential between two bilayers^13^, which includes electrostatic, Van der Waals and hydrophobic interaction potentials (Supplementary Fig. 3). Comparison of the theoretical model with previous work (see Supplementary Note 1) indicates that bilayer thinning and hydrophobic interactions lead to fusion^13^. Separation after slow hemifusion of the bilayers leads to an adhesion force of *F*_ad_/*R*=–24±3 mN m^−1^, which can be converted to adhesion energy using the Johnson–Kendall–Roberts (JKR) model^24^,^25^, *W*_ad_=*F*_ad_/1.5*πR*=–5.0±0.7 mJ m^−2^. The interleaflet hydrophobic attraction energy is much smaller compared with the expected value for fully hydrophobic surfaces –100 mJ m^−2^ (ref. 26), which is because of segregation of curvature-favouring lipids (that is, DOPC) at the boundaries of the stalks, as discussed later. After FR2, a third FR on the same contact revealed that the steric hard wall was shifted down from 8.7 to 5.6 nm, slightly larger than the thickness of a single bilayer. However, during separation, no adhesion force was measured, indicating that lipid molecules partially mended the damaged bilayers.

After FR3, a high compression (*F*/*R*=1,150 mN m^−1^) experiment was performed (Supplementary Fig. 4) with *t*_c_=23 h. High compression induces *fast* hemifusion, which was completed in 1 h (from Fig. 4g to j). Immediately after compression, the central, previously hemifused region (during FR2) exhibited the thickness of a single bilayer, while the edge region had a thickness of two bilayers (Fig. 4g). Within 1 h, the edge also completely hemifused to a single bilayer (Fig. 4j). On separation after *t*_c_=23 h, *W*_ad_=–22.9 mJ m^−2^(*F*_ad_/*R*=–108 mN m^−1^) was measured—still smaller than the expected value of –100 mJ m^−2^ (ref. 26), but similar to previously measured *W*_ad_ of –20 mJ m^−2^ between two hemifused *trans*-azobenzene trimethylammonium bromide bilayers^13^. To check whether the hemifusion of a pristine bilayer (which was not fused before) also initiates from the centre of the contact, we repeated the high compression experiment without an initial low compression (Supplementary Fig. 5); again, hemifusion started at the centre (see Supplementary Movie 1). This behaviour indicates that hemifusion initiates because of the maximal local pressure at the centre of the JKR contact. The measured adhesion energy was also similar to what was measured in FR4 (Supplementary Fig. 4).

### Lipid domain rearrangements during hemifusion

From the initial membrane–membrane contact to the complete hemifusion of lipid bilayers, significant reorganization of lipid domains (L_o_) was observed as displayed in Figs 3c and 4f,i,l. Both low and high compressions display similar domain reorganization behaviour, although the timescale of hemifusion is different. The reorganization of lipid domains (L_o_) during high compression was investigated in detail (see Fig. 5, Supplementary Movies 2 and 3), and can be summarized as follows: (1) first, the L_o_ phase is depleted from the contact (in at least one bilayer), rapidly diffusing out and forming a dark (L_o_–L_o_) rim surrounding the bright (L_d_–L_d_) and grey (L_d_–L_o_) contact and also slowly disappearing by lipid molecules mixing with the L_d_ phase. The average L_o_ phase disappearance rate was ∼100 μm^2 ^min^−1^ (see Supplementary Fig. 6a) after applying a constant load (*L*=23 mN) and at *t*_c_=14 min, the L_o_ phase was fully depleted from the contact in at least one bilayer. (2) The hemifusion of two lipid bilayers initiates near the centre of the contact, where the stress is highest and the two L_d_ phases (L_d_–L_d_, which has the lowest-energy barrier for fusion) were in contact. The hemifused region reveals itself as a dark spot inside the contact, which is surrounded by a bright (L_d_–L_d_) rim. (3) The hemifused area propagates and grows logarithmically with *t*_c_ (see Supplementary Fig. 6b) to the size of the initial contact (or even slightly larger because of higher adhesion), which results in completely hemifused bilayers. The final image shows the dark ellipsoidal (or circular) contact with a bright (L_d_–L_d_) rim surrounding it.

## Discussion

Under low compression, hemifusion took almost 19 h to complete, while under high compression the bilayers hemifused in 2.5 h (1 h for the previously fused bilayers, Fig. 4; and 2.5 h for the pristine bilayers Fig. 5 and Supplementary Movies 2 and 3). The dynamics of domain rearrangements contribute to slow hemifusion. Localization of the L_d_ phase at the contact lowers the energy barrier for hemifusion because of a larger area per molecule exposing more hydrophobic groups^13^,^14^. The rate of the L_o_ phase depletion is proportional to the applied load. Nevertheless, the hemifusion processes here are much slower than *in vivo* during membrane trafficking that takes milliseconds to minutes^10^. The difference in the timescale of fusion originates from the differences in the energy barrier to fusion, which is significantly affected by the membrane curvature diameter (centimetres versus tens of nanometres), the mobility of lipids (supported versus free-standing), and the temperature (room temperature versus body temperature).

The L_d_ phase, which forms within 2.5 h at the edge of the stalk, as indicated by the bright rim in Fig. 5, is stable (or at least metastable) for more than 12 h, so long as the bilayers are kept under pressure in the hemifused state, that is, not detached from each other. If the bright rim observed after the hemifusion was just a pile-up of lipids (which includes dye-containing lipids), the thick pile-up would be easily observable as a deformation of fringes of equal chromatic order (FECO). However, the FECO show no noticeable deformation (Fig. 4j); thus, we conclude that the bright rim is indeed a selective localization of the L_d_ phase. Previous studies on lipid membranes^18^,^27^ have shown that BSM-rich membranes (L_o_) have a higher bending rigidity compared with the DOPC-rich membranes (L_d_). In order to lower the bending energy, the L_d_ phase is enriched in high-curvature membrane regions, as observed by the formation of the bright rim around the edge of the contact region (Fig. 5). When the hemifused bilayers are separated and relaxed, the bright L_d_ phase rim becomes delocalized and disappears (Supplementary Fig. 7), providing further evidence that the bright L_d_ phase rim stabilizes the energetically unfavourable stalk edge.

Here using the FL-SFA, domain reorganization has been imaged in real time during hemifusion. The migration of the L_d_ region to the edge of the contact zone, combined with the small measured values for *W*_ad_, shows that the domains (L_o_) rearrange into their lowest-energy state during fusion. The fusion rate (and rate of rearrangement of the domains) is much faster at larger applied pressures, suggesting that the extra energy input into the system activates faster mixing of the leaflets. These results highlight the role and molecular mechanisms of lipid domains (L_o_) during hemifusion of model membranes, indicating that domains can rearrange to decrease the energy barrier and increase the rate of fusion in membrane processes.

The use of FL-SFA can be extended further to monitor dynamic transformations in systems where lipid domains are likely or known to occur (including pathological biological membranes) and gather previously unobtainable fundamental insights. In addition to model membrane systems, the FL-SFA has a wide range of potential applications for studying dynamic rearrangements/adsorption and forces of various interacting/noninteracting materials during and after confinement. These materials could include surfactant mono- and bilayers, biomolecules, colloidal particles, nanoparticles, polymers and smart materials. In these natural and engineered systems, close proximities and dynamic changes often occur, which can now be studied in greater detail using the FL-SFA.

## Methods

### Materials

Lipids used in this study that were purchased from Avanti Polar lipids (Alabaster, AL) were as follows: DPPE (16:0, Powder), DOPC (18:1, Chloroform), BSM (predominant 18:0, Porcine, Chloroform) and CHOL (ovine wool, ≥98%). For the fluorescence imaging, Texas Red 1,2-Dihexadecanoyl-sn-Glycero-3-Phosphoethanolamine, TR-DHPE was purchased from Invitrogen (Carlsbad, CA). DPPE was dissolved in a solvent, which is a 3:1 (vol/vol) mixture of chloroform (Sigma-Aldrich, CHROMASOLV Plus for HPLC, purity ≥99.9%) and methanol (Sigma-Aldrich, CHROMASOLV Plus for HPLC, purity ≥99.9%) at a final concentration of 1 mg ml^−1^. DOPC, BSM and CHOL were mixed in a 1:1:1 (mol/mol) solution at a final concentration of 1 mg ml^−1^ in chloroform. A trace amount (1 wt%) of TR-DHPE was added to the mixture for imaging purposes. All lipids were stored in a deep freezer (−50 °C) until use. Buffer salts were purchased from Sigma-Aldrich (St Louis, MO), mixed and dissolved in Milli-Q water (Millipore, Billerica, MA) at final concentrations of 100 mM Sodium nitrate (ReagentPlus, purity ≥99.0%), 10 mM Tris(hydroxymethyl)aminomethane (ACS reagent, purity ≥99.8%) and 2 mM Calcium nitrate tetrahydrate (purity ≥99.0%) at a pH of 7.5.

### FL-SFA

A standard SFA2000 system (SurForce LLC, Santa Barbara)^28^ was modified in order to enable simultaneous fluorescence imaging with the force–distance profiling. The two most critical modifications were (i) replacing the reflective layer of silver with a hard quarter wave plate coating to allow for wavelength-dependent specific reflective and transmission regions (see below and Supplementary Fig. 8) and (ii) modifying the optical paths to allow for the necessary filters and mirrors for fluorescence imaging.

**Figure 1:**
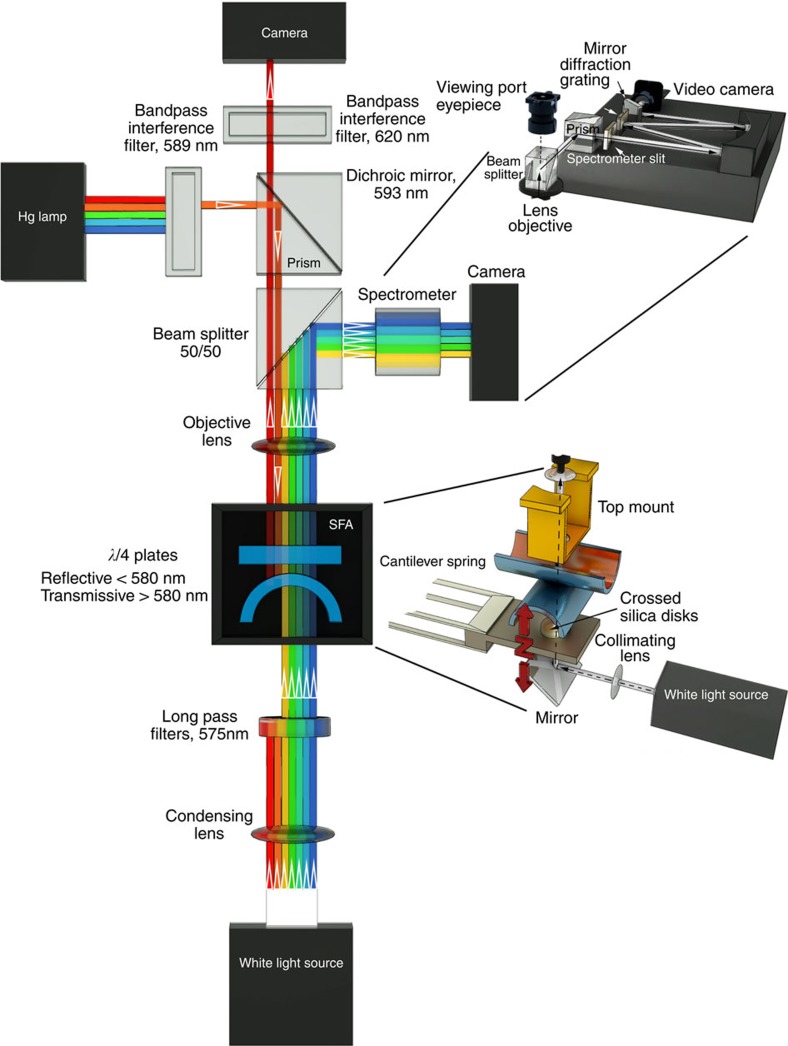
Schematic of the FL-SFA. The mica surfaces inside the SFA (centre) are back-coated with a quarter wave plate that allows for (i) reflection below 580 nm wavelength of light used for the multiple beam interference in standard SFA measurements, and (ii) transmission above 580 nm wavelength of light used for fluorescence microscopy. The quarter wave plate coating and the beam splitter allow for simultaneous measurements of SFA data and fluorescence imaging with only a small effect on the resolution or performance of both methods.

**Figure 2:**
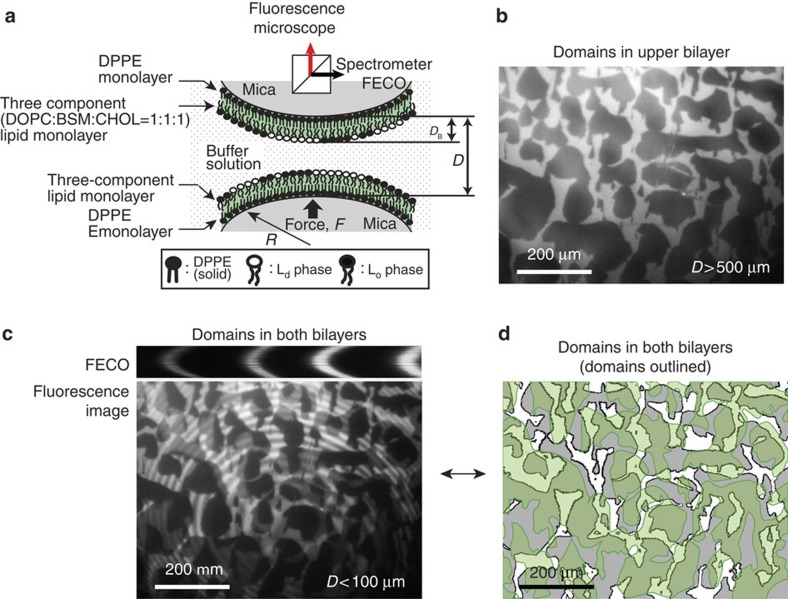
Schematic of the experimental set-up and obtained lipid domain images. (**a**) Two asymmetric bilayers deposited on mica surfaces using LB deposition technique and (**b**–**d**) images of lipid domains obtained with FL-SFA. (**b**) Lipid domains of only upper bilayer, (**c**) lipid domains in both bilayers and (**d**) outlined and shaded lipid domains.

**Figure 3:**
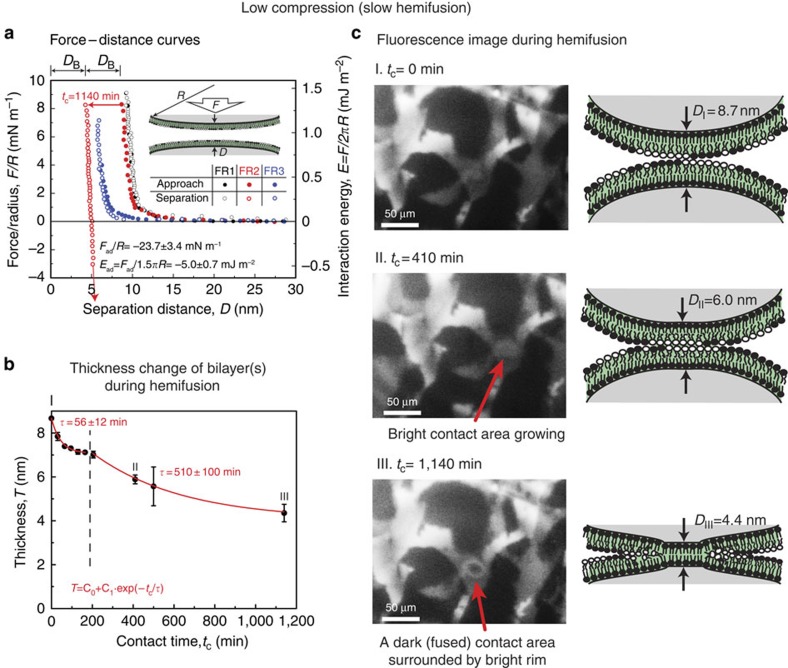
Low compression FL-SFA results. (**a**) Force–distance (*F*–*D*) curve measured between two bilayers performed under low compression (*F*/*R*=8 mN m^−1^). The error value is the s.d. from the repeats of at least four experiments performed with different bilayers and contacts. (**b**) The bilayer thickness change, during *slow* hemifusion of two bilayers during a contact time of 1,140 min and (**c**) lipid domain reorganization during hemifusion.

**Figure 4:**
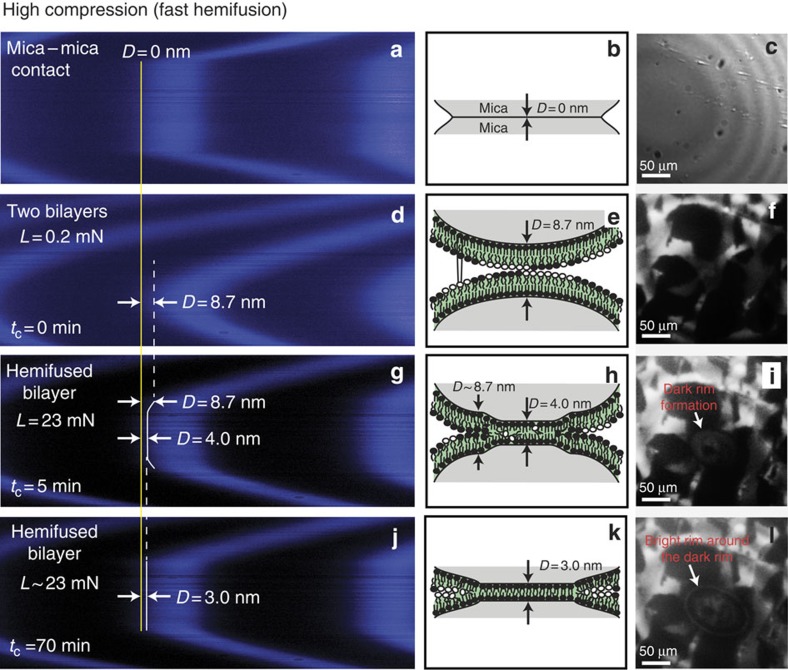
Optical and FL imaging of compressed bilayers in the SFA. Simultaneous monitoring of the FECO (**a**,**d**,**g**,**j**), normal optical microscope showing Newton's rings (**c**) and lipid domain localization (**f**,**i**,**l**) using FL-SFA before and during the contact time of FR4 (see Fig. 3b), and their schematics (**b**,**e**,**h**,**k**). (**a**–**c**) Bare mica–mica contact; (**d**–**f**) two bilayers before FR2; (**g**–**i**) hemifused bilayers right after high compression; and (**j**–**l**) hemifused bilayers at *t*_c_∼70 min.

Figure 1 shows the FL-SFA set-up where the fluorescence light is illuminated from above and the white light for force–distance profiling is from below. A longpass filter of 575 nm in front of the white light source minimizes its interference with the fluorescence imaging. The remainder of the white light is passed through the mica surfaces with the quarter wave plate-coating (see below and Supplementary Fig. 8a). Emerging multiple beam interfering (MBI) light from the cavity created by the reflective quarter wave plate-coating at the backside of the mica surfaces is guided via a 50/50 beam splitter to a spectrometer, where MBI light is diffracted into FECO. The FECO are recorded with a Princeton CoolSNAP CCD camera. The FECO provide information of both absolute separation distances between the two mica surfaces and the profile of the apposing curved surfaces^28^. The lower surface is mounted to a double cantilever spring with a known spring constant *k*, which allows for accurate force measurements.

The fluorescent dye Texas Red used in this study has an absorption peak at 589 nm. A mercury lamp with a short band filter at 589 nm wavelength (10 nm wavelength width) provides the excitation light for the fluorescence imaging. A dichroic mirror at 593 nm wavelength allows for reflective mode imaging as it reflects the excitation light from the Hg lamp and transmits the emission light from the fluorescent dye. The lipid sample with Texas Red (see preparation below) fluoresces with a peak around 615 nm. The emission light from the fluorescent dye is passed through the dichroic mirror and a short pass filter at 620 nm wavelength (10 nm wavelength width) and then recorded with a Hamamatsu Orca-R2 CCD camera.

The boundary of 580 nm wavelength of light for the quarter wave plate coating is chosen for fluorescence imaging using Texas Red. This boundary can easily be shifted to allow for other types of fluorophores. The numerical aperture of the objective lens is 0.27 for our set-up.

### Substrate preparation

Atomically smooth mica surfaces of thickness 2–4 μm were freshly cleaved in a laminar flow hood and immediately attached to a larger and freshly cleaved backing sheet of mica for storage, which prevents the mica surfaces from contamination^28^. The back side of the mica surfaces was coated with quarter wave plate using the Ion Beam Deposition technique. Alternating layers of Ta_2_O_5_ and SiO_2_ with number of layers together with thicknesses as shown in Supplementary Fig. 8b provide a wavelength-specific reflective hard coating. Supplementary Fig. 8c shows the calculated reflectivity of the mica-quarter wave plate system. Incident light of wavelength below 580 nm is reflected, while above 580 nm light is transmitted through the quarter wave plate coating. The mica surfaces were glued (EPON 1004F, From Exxon Chemicals) with the quarter wave plate coating down on cylindrical silica disks. The surfaces were placed in a cross cylindrical configuration in the SFA, which corresponds to a sphere-on-flat configuration.

### Substrate preparation and transfer to an SFA

Lipid bilayers were deposited on the mica substrates (prepared as above), using the LB deposition technique^29^ at room temperature. First, the prepared mica surfaces were dipped into Milli-Q water using a dipper attached to the LB trough. The air–water interface was carefully cleaned with a suction pipette to remove any existing dust particles at the interface. As a first layer, 100 μl of 1 mg ml^−1^ DPPE solution was slowly spread on the air–water interface and the solvent was allowed to evaporate for 15 min. Starting from a total area of ∼755 cm^2^, the DPPE monolayer was compressed slowly (10 cm^2 ^min^−1^). After reaching the target surface pressure, *Π*=35 mN m^−1^, which gave a molecular area of *A*=42 Å^2^ (Supplementary Fig. 1a), the mica surfaces were raised at 1 mm min^−1^. After the first monolayer deposition, the substrates were stored in vacuum for 12 h. Having the solid-like DPPE as the supporting layer is advantageous as follows: (i) it eliminates the long-range Helfrich undulation force, which may affect distinguishing between the other forces (for example, electrostatic, steric and hydrophobic); (ii) it greatly reduces the mobility of the free (unstressed) lipid domains (L_o_), which is advantageous for focusing only on the lipid domains at the contact and (iii) interleaflet domain coupling can be excluded since the DPPE monolayer at room temperature is in the solid phase, and also not expected to phase separate. As a second layer, 100 μl of 1 mg ml^−1^ 1:1:1 (mol/mol) DOPC:BSM:CHOL+1 wt% TR-DHPE mixture was spread on to a previously prepared buffer. The deposition conditions and compression and dipper velocities for the second (outer) DOPC:BSM:CHOL monolayer were the same as for the supporting (inner) DPPE monolayer except that the dipping direction was reversed and the target surface pressure was *Π*=30 mN m^−1^ (*A*=41 Å^2^ per molecule, Supplementary Fig. 1b). After the deposition, without exposing the surfaces to air, the surfaces were placed into small glass Petri dishes filled with buffer. The Petri dishes were transferred to the SFA chamber that was filled with degassed saturated lipid solution (buffer in contact with lipid crystals for 12 h and degassed 2–3 h with a vacuum pump), and the surfaces were mounted to upper and lower disk holders for the experiments.

### FL-SFA experiments

The FRs were performed statically using a fine control motorized micrometre, with step sizes of 2–3 nm and equilibration time of 5–10 s at each point. During the high compression experiment, after approaching surfaces to an *F*/*R* value of 8 mN m^−1^ with a fine control motorized micrometre, a medium control micrometre was used to compress the surfaces even further (30 μm, which corresponds to *F*/*R*=1,150 mN m^−1^). During the separation after high compression, the medium control micrometre was used to separate the surfaces and measure adhesion force. FRs were performed in the order as mentioned in the main text, followed by four or more repeat experiments (see Supplementary Figs 2, 4 and 5) with different bilayers and/or contacts. Fluorescence imaging was performed simultaneously with FRs, especially focusing on the images before compression, right after compression, during hemifusion and after separation.

**Figure 5:**
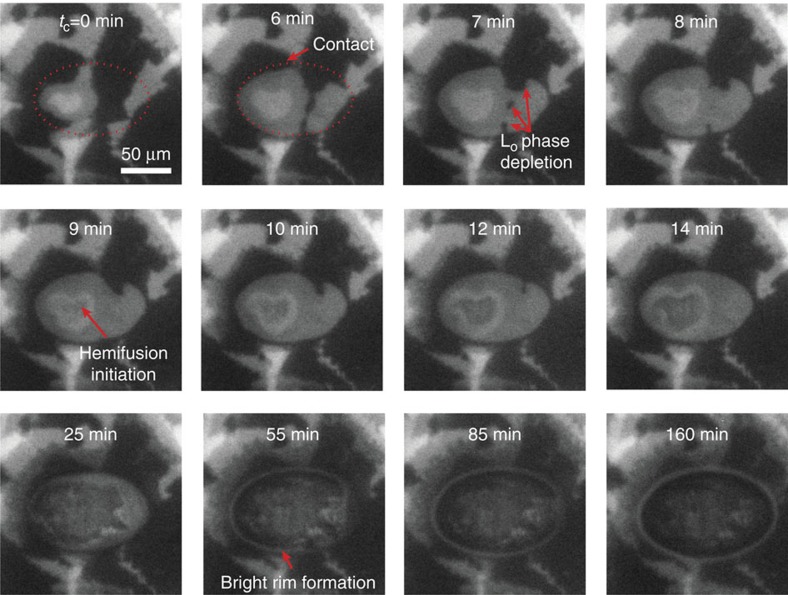
Fluorescence image of a contact (Sample no. 2, contact no. 1 from Supplementary Fig 2) as a function of time, immediately after high compression (*F*/*R*=1,150 mN m^−1^).

During the waiting time (under compression), FECO and fluorescence images are continuously monitored (when drastic fast changes in the bilayer images are observed; Fig. 5, Supplementary Movies 2 and 3), or intermittently imaged every 30–60 min (when slow changes are observed). Optimized fluorescence images required 5–10 s of exposure time. During the time *between* imaging, the mercury light for fluorescence imaging and white light for FECO imaging were blocked to protect the fluorophore from photobleaching.

All experiments were performed at room temperature.

## Additional information

**How to cite this article:** Lee, D.W. *et al.* Real-time intermembrane force measurements and imaging of lipid domain morphology during hemifusion. *Nat. Commun.* 6:7238 doi: 10.1038/ncomms8238 (2015).

## Supplementary Material

Supplementary Information Supplementary Figures 1-8, Note 1 and Supplementary References.

Supplementary Movie 1 FECO video showing hemi-fusion of lipid bilayers (Supplementary Fig. 5). Hemi-fusion started from the center and propagated to the edge. White, yellow, and red lines indicate D=2DB, D=DB, and D=0 nm, respectively. The video was accelerated 20 times faster than real time.

Supplementary Movie 2 Continuous video for Figure 5, from 0 to 8 minutes. Slow replacement of Lo phase to Ld phase has been imaged. The video was accelerated 20 times faster than real time.

Supplementary Movie 3 Reorganization of the lipid domains during hemifusion (sample #2, contact #2). The video was accelerated 20 times faster than real time.
