# Supplementary material for: Real-time intermembrane force measurements and imaging of lipid domain morphology during hemifusion
Source: Nat Commun. 2015 May 26;6:7238. doi: 10.1038/ncomms8238 (PMC4455132; doi:10.1038/ncomms8238)
Supplement: Supplementary Information — Supplementary Figures 1-8, Note 1 and Supplementary References. [file ncomms8238-s1.pdf]

Supplementary Figures

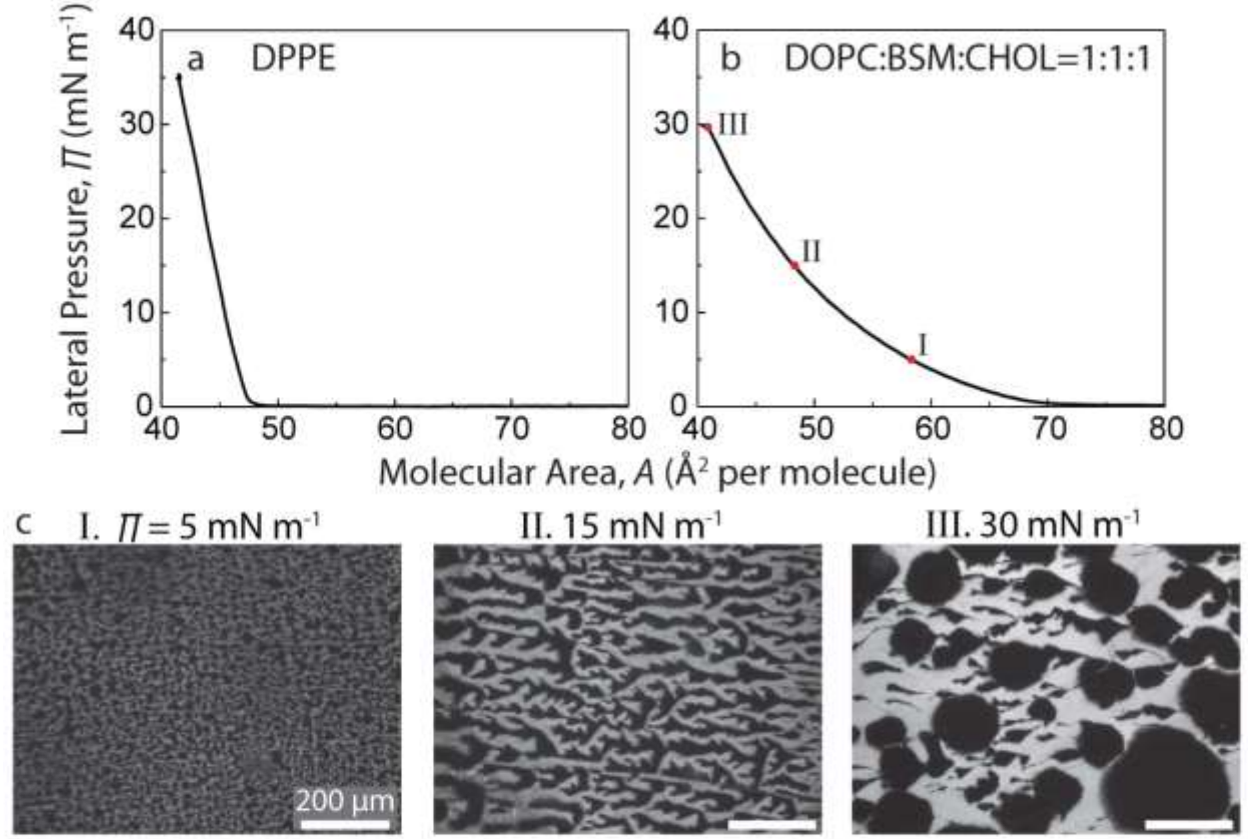

Supplementary Figure 1. Lateral pressure (*Π*) - Molecular Area (*A*) isotherm of (**a**) DPPE, and (**b**) DOPC:BSM:CHOL = 1:1:1 mixture. (**c**) Fluorescence images showing lipid domains in DOPC:BSM:CHOL=1:1:1 mixture which was LB deposited at three different pressures (5, 15 and 30 mN m^{-1}) on to DPPE monolayer.

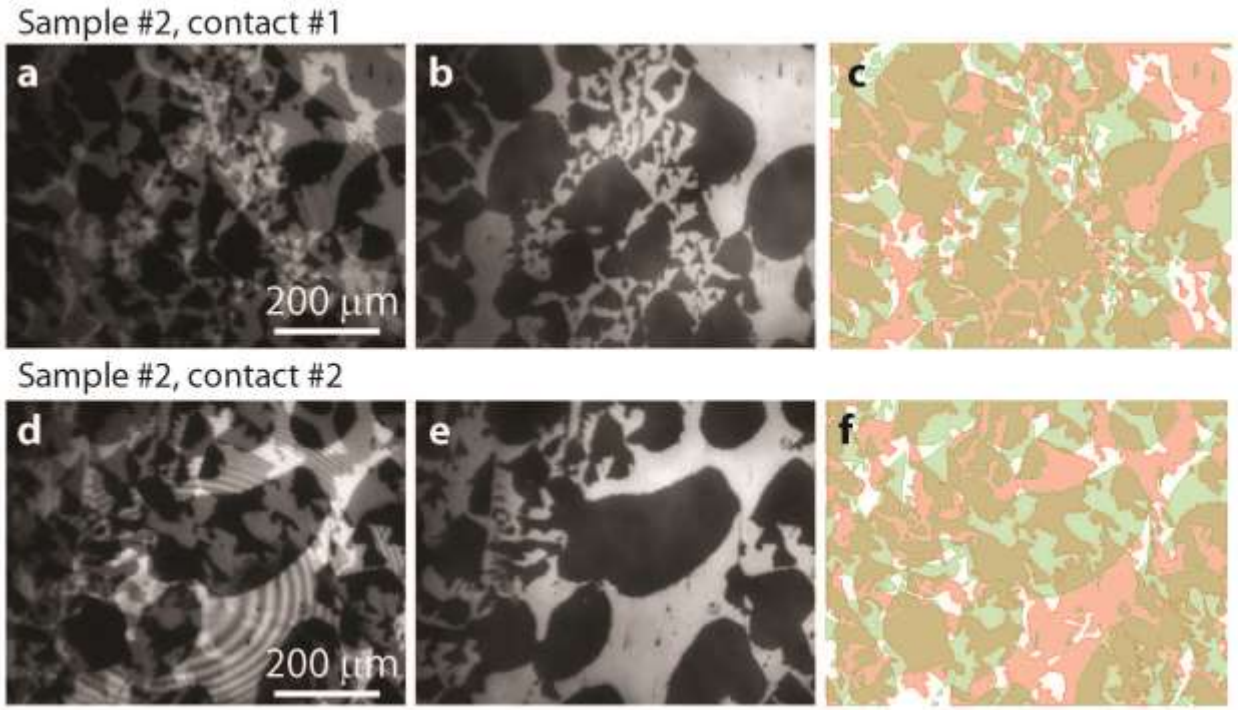

Supplementary Figure 2. Domains in bilayers (repeat experiments); (**a, d**) overlapped domains, (**b**, **e**) domains in upper bilayer, and (**c**, **f**) distinctively pseudo-colored domains (upper domains as green, and lower domains as red). The ring formations observed in **a** and **d** are Newton's rings, where the center of the rings is the point of closest approach between the two curved surfaces.

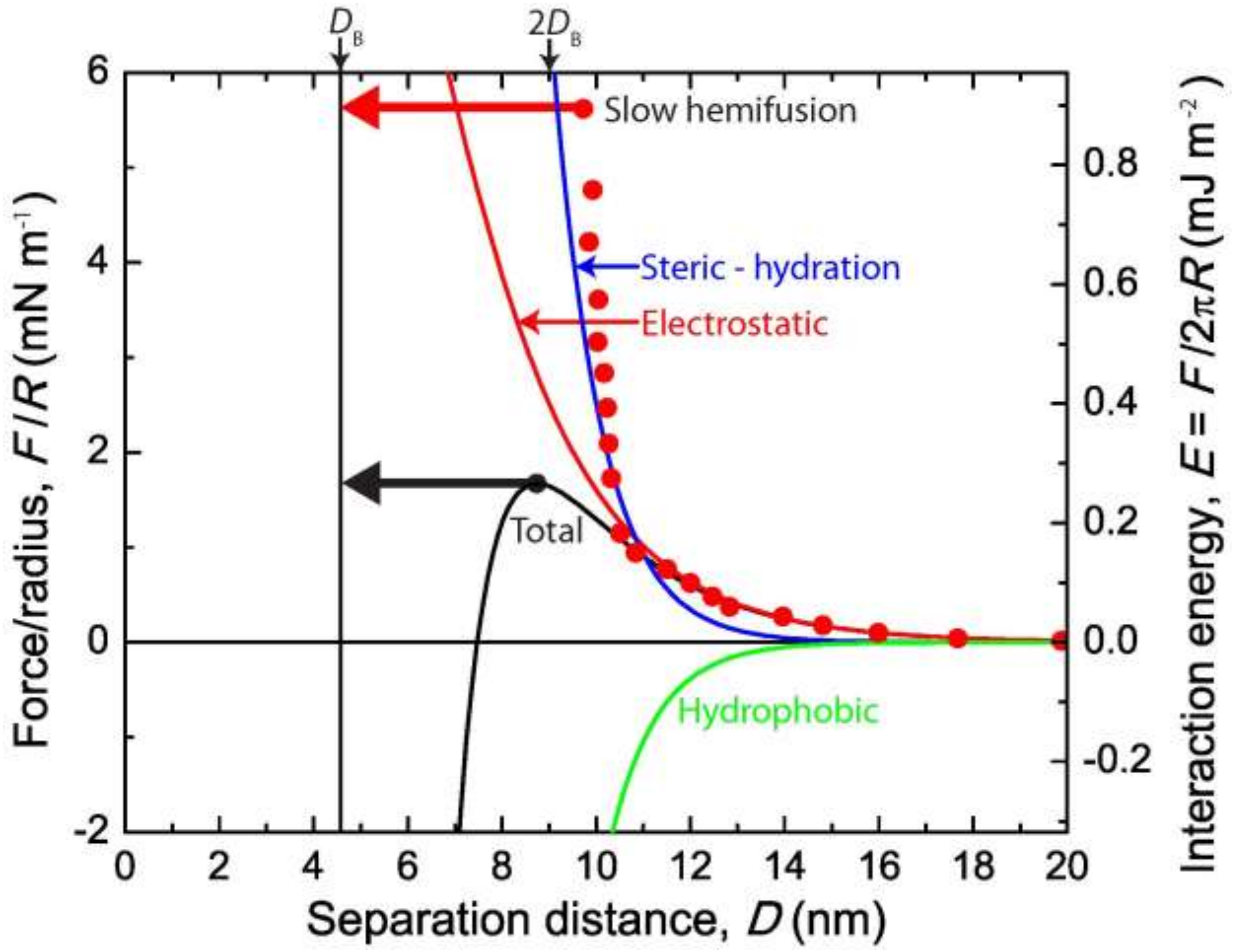

Supplementary Figure 3. **Measured *F-D* curve during hemifusion of bilayers (red points, also shown in main text, FR2 in Fig. 3) and theoretical analysis for hemifusion.** The overall model (black curve) includes contributions from hydrophobic interactions (green curve), steric-hydration repulsion (blue curve), and electrostatics (red curve). The model predicts hemifusion at the turning point in the curve (where it changes from attractive to repulsive), indicated by the thick black arrow. The thick red arrow is the point at which slow hemifusion was measured (see Fig. 3). The theoretical curve predicts the "equilibrium" fusion barrier, while the measured barrier reflects the slow rearrangements discussed in the main text.

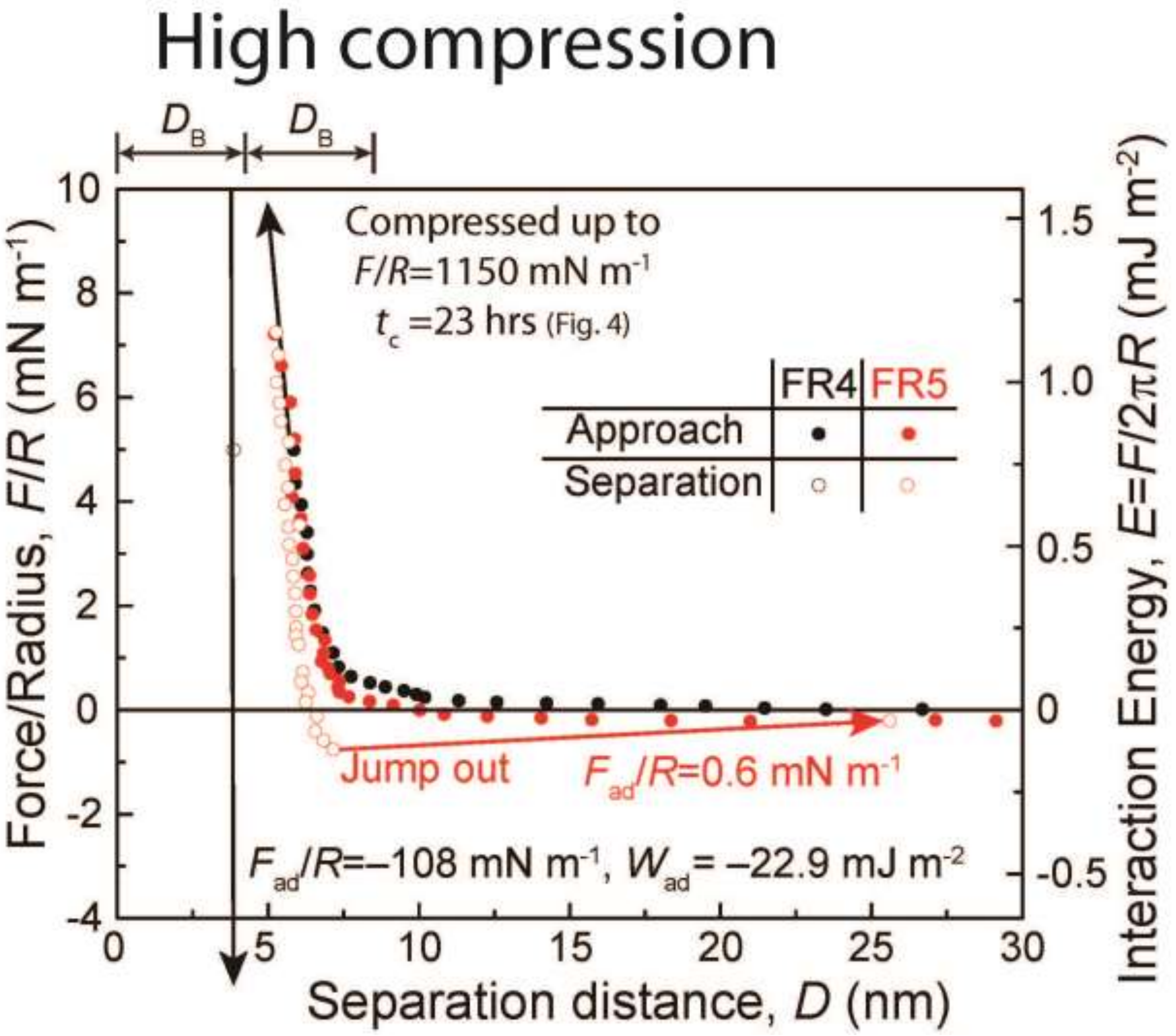

Supplementary Figure 4. High compression induced hemifusion (FR4) and consequent force runs (FR5) performed after FR3 (Fig. 3a). Hemifused bilayers resulted in adhesion energy, W_{ad}=-22.9 mJ m^{-2}.

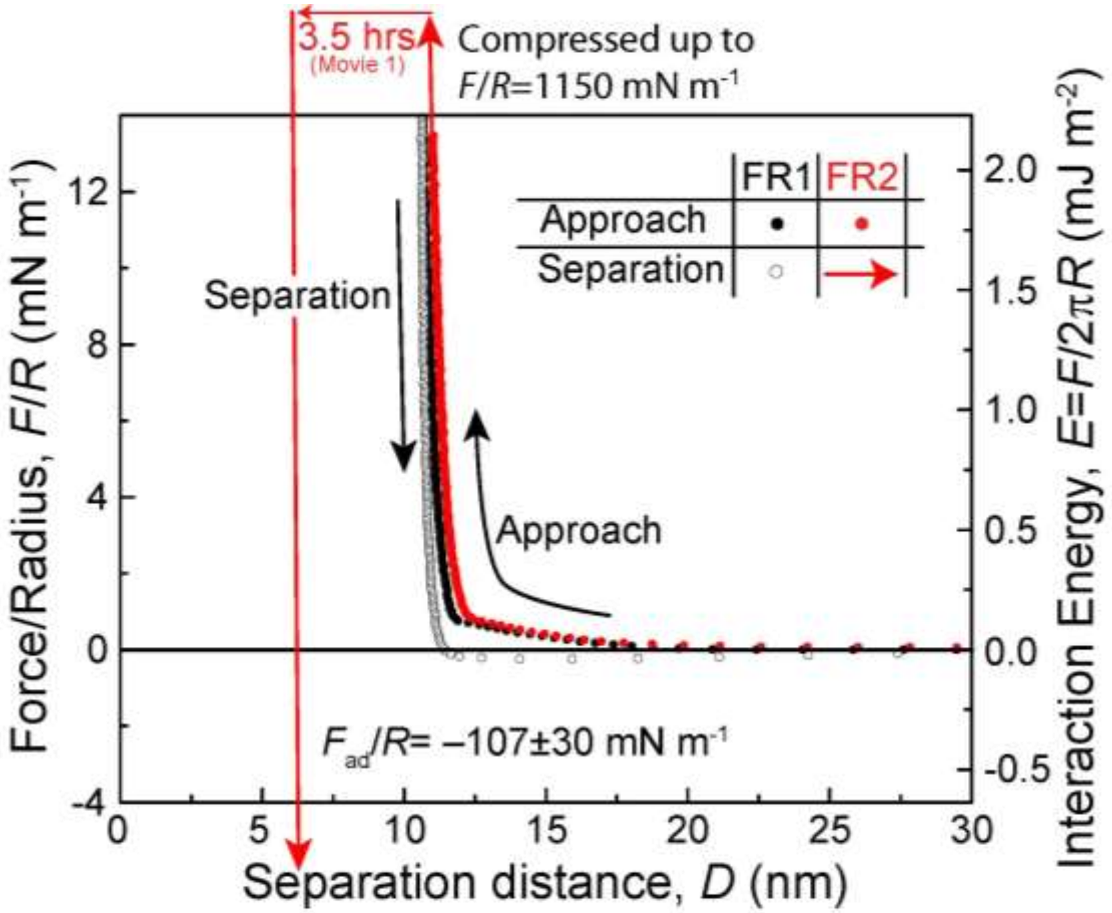

Supplementary Figure 5. High compression (FR2) control experiment *without* the addition of fluorophores and without the initial hemifusion due to low compression. Similar force curves were observed as in Figure 3a except that there is hysteresis between approach and separation possibly due to the control experiment being performed under dynamic loading and unloading conditions.

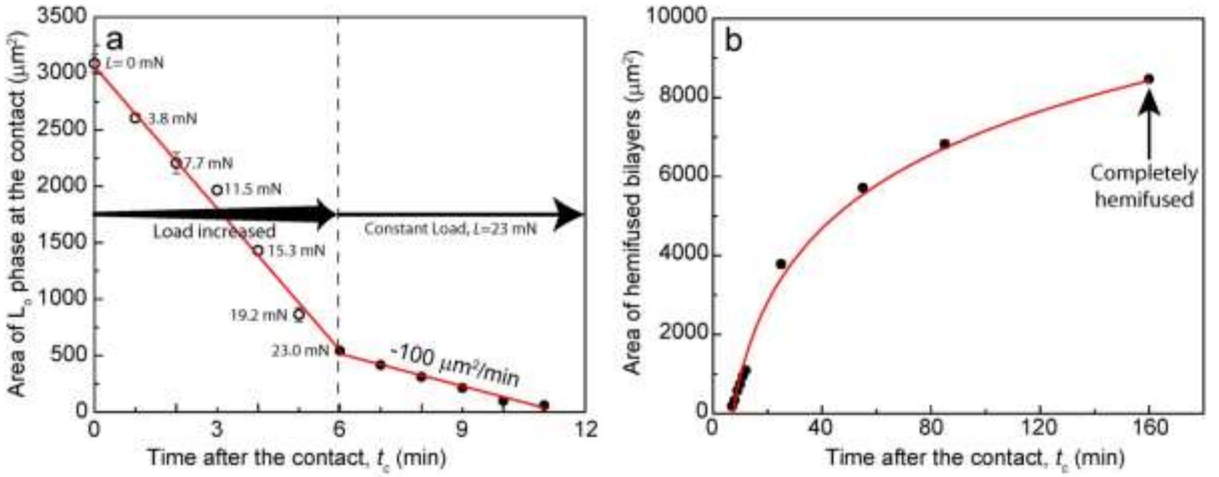

Supplementary Figure 6. Area change in (a) L_o phase and (b) hemifused bilayers with contact time (t_c), analyzed from Figure 5. Open circles indicate the load increasing regime, and closed circles indicate constant load regime.

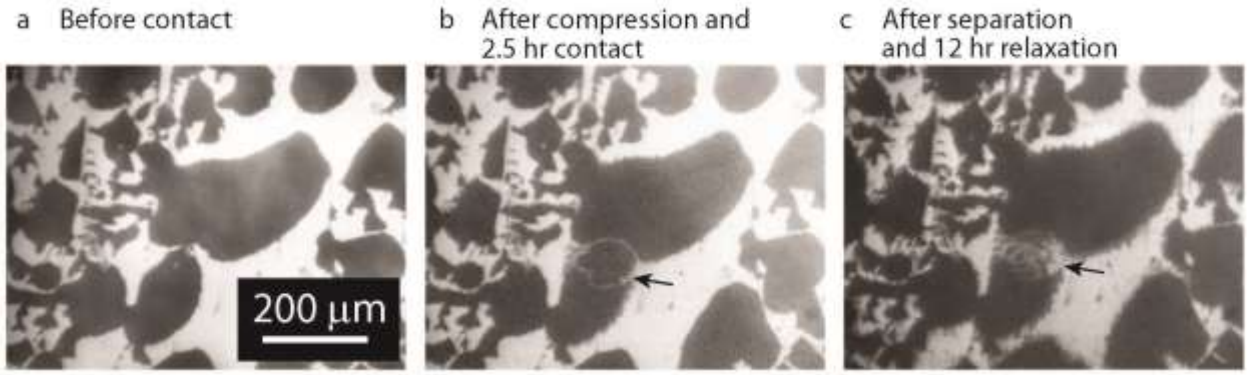

Supplementary Figure 7. Domains at the upper surface of Sample #2, contact #2, (**a**) before compression, (**b**) after 2.5 hr compression (note the contact near the center of the image indicated with arrow has dark – L_o state with a white rim – L_d state), and (**c**) after 12 hr relaxation showing rearrangement of the previous contact area and roughening of other domain boundaries.

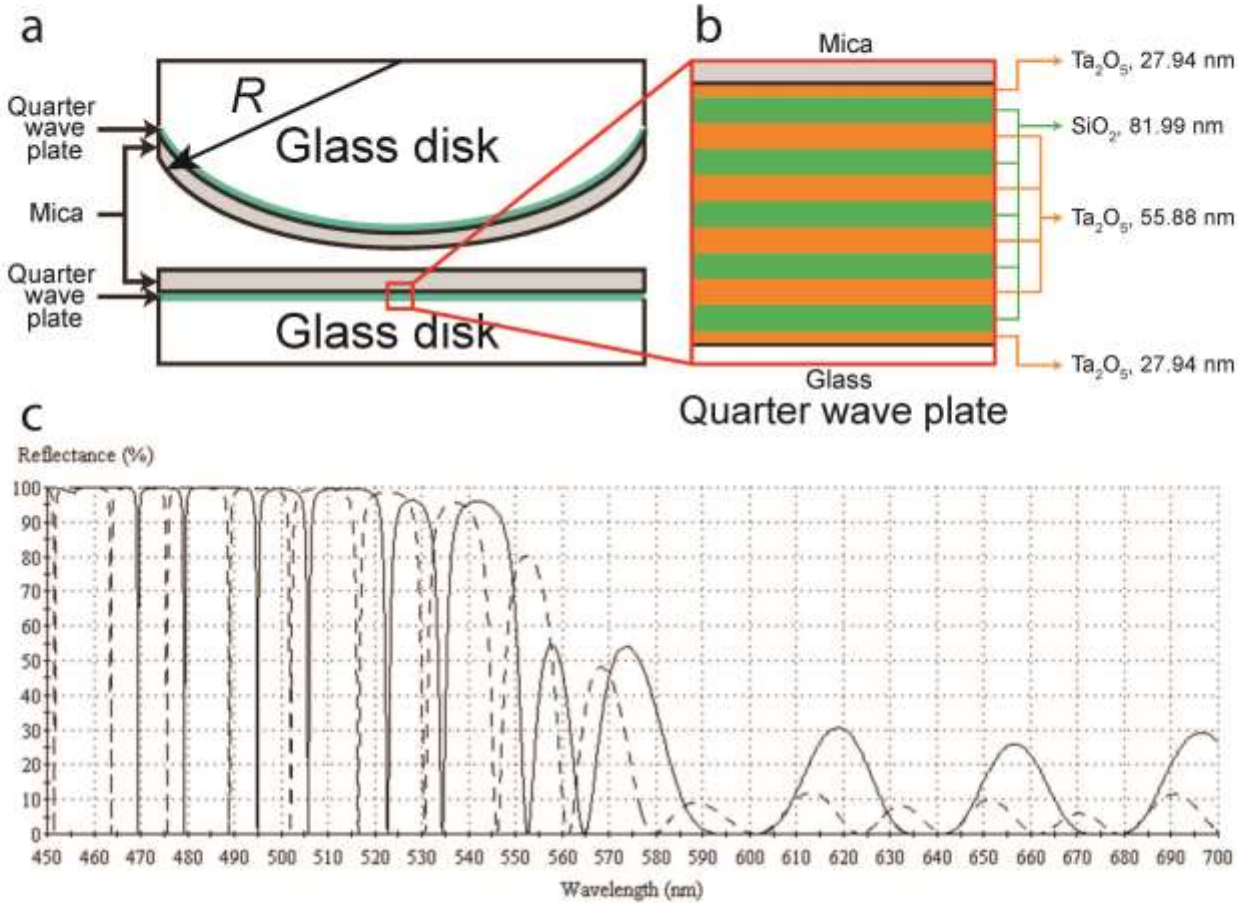

Supplementary Figure 8. Schematic of quarter wave plate and its reflectance plot as a function of wavelength. (a) The mica sheets are glued with the deposited quarter wave plate down on the glue layer. **(b)** Eleven alternating layers of Ta_2O_5 and SiO_2 form the quarter wave plate, and were deposited using Ion Beam Deposition at a rate of 1.2 Å s^{-1} and 0.8 Å s^{-1}, respectively. **(c)** Using the Essential Macleod software with quarter wave plates with layer thicknesses given in **b**, the solid line shows that the reflectance through the whole system (quarter wave plate – 3 µm mica – 100 nm air – 3 µm mica – quarter wave plate) is high up to 580 nm and low below that value (and highly transmittive above 580 nm). The dotted line shows the reflectance from the same stack, but with a 10 nm air gap instead of 100 nm. The transition from reflective to transmittive at 580 nm was confirmed using an Optical Film Thickness tool (Filmetrics) and the Multiple beam interference technique.

Supplementary Note 1

Theoretical analysis of hemifusion

As measured previously[1,2], hemifusion occurs when the hydrophobic core of the bilayer is exposed, causing attractive hydrophobic forces to overwhelm repulsions due to electrostatics and steric forces. Therefore, the theoretical analysis presented here uses equation 1 shown below that includes contributions from elastic deformations of the membrane, hydrophobic, electrostatic, and steric-hydration interactions, which was previously derived for light-responsive bilayers[1].

$$W_{\text{total}} = 2\gamma_i \frac{{a_0}^2}{a(d)^2} - 2\gamma_i - 2\gamma_i \left(1 - \frac{a_0}{a(d)}\right) e^{-d/D_{\text{H}}} + \frac{C_{\text{ES}}}{a(d)} e^{-\kappa d} + \frac{C_{\text{SHR}}}{a(d)} e^{-d/D_{\text{SHR}}} \tag{1}$$

This equation is derived fully in previous work, but briefly, the first term accounts for the elastic bending energy of the membrane, the second term is the reference state energy at D → ∞, the third term is the hydrophobic attraction, the fourth term is the electrostatic repulsion, and the final term is the steric hydration repulsion. In these experiments, representative values are used for the equilibrium headgroup area, a_0 = 50 Å^2, interfacial tension γ_i = 50 mJ m^{-2}, hydrophobic decay length D_H = 1 nm, and steric hydration decay length D_SHR = 1 nm. Fitted parameters are C_ES = 1.3x10^{-21} J, κ^{-1} = 2 nm, and C_SHR = 3x10^{-20} J. The area per molecule is a function of distance that arises from minimizing the energy and is calculated from

$$a(d) = a_0 (1 - e^{-d/D_{\text{H}}})^{-1/2} \tag{2}$$

The theoretical energy is calculated as a function of bilayer separation distance, *d*. The bilayer thinning is accounted for by plotting the calculated energy as a function of mica-mica separation, *D*, where $D = d + 2T$, where $T = v_0/a(d)$ and $v_0 = a_0 \cdot L_0$. Thus the thickness of the bilayers varies as a function of separation distance, and the final hardwall has the thickness of a single bilayer. For full details on the fitting, the reader is referred to our earlier work[1].

As shown in Figure S3, the theoretical analysis described above quantitatively accounts for the long range and short range forces during bilayer hemifusion. The black curve shows the theoretical curve while the red points are the same experimental points shown in the main text (Fig. 3). The theoretical breakthrough occurs at a distance of about *D* = 8.8 nm, close to the thickness of two bilayers, $2D_\text{B}$ = 9 nm. The fitted parameters are similar to those in previous work, indicating that the mechanism for fusion is likely the same, i.e., compression results in thinning and spreading of the bilayer, resulting in hydrophobic pores and hydrophobic attraction between the bilayer interiors on opposite surfaces. The measured force barrier is larger than the predicted barrier at equilibrium, reflecting that the hemifusion processes of compressing the bilayers and molecular rearrangements within the bilayer occur slowly and dynamically.

Supplementary References

1. Donaldson, S.H., Lee, C.T., Chmelka, B.F. & Israelachvili, J.N. General hydrophobic interaction potential for surfactant/lipid bilayers from direct force measurements between light-modulated bilayers. *Proceedings of the National Academy of Sciences of the United States of America* **108**, 15699-15704 (2011).
2. Helm, C.A., Israelachvili, J.N. & McGuiggan, P.M. Molecular mechanisms and forces involved in the adhesion and fusion of amphiphilic bilayers. *Science* **246**, 919-922 (1989).
